# Larval fish counteract ram and suction to capture evasive prey

**DOI:** 10.1098/rsos.220714

**Published:** 2022-11-02

**Authors:** Irvin Chang, Daniel K. Hartline, Petra H. Lenz, Daisuke Takagi

**Affiliations:** ^1^ Department of Mathematics, University of Hawai'i at Mānoa, 2565 McCarthy Mall, Honolulu, HI 96822, USA; ^2^ Pacific Biosciences Research Center, University of Hawai‘i at Mānoa, 1993 East-West Road, Honolulu, HI 96822, USA

**Keywords:** hydrodynamic stealth, copepod, larval fish, predator–prey interaction, suction feeding, ram feeding

## Abstract

A simple hydrodynamic model of predator–prey interactions between larval clownfish and copepod prey is used to elucidate how larval fish capture highly evasive copepods. Fish larvae are considered to be suction feeders; however, video observations revealed that successful captures by clownfish larvae were preceded by rapidly accelerating lunges (ram), while the role of suction to draw prey into the fish's mouth was less clear. Simulations were made of the fish's strike, varying strengths of ram and suction to characterize optimal strategies for copepod capture given known evasive capabilities. Our results suggest that, contrary to expectations, suction feeding is dominant only in older larvae, whereas ram feeding is the dominant mode for early larvae. Despite the relatively weak suction produced by smaller larvae, it still plays a crucial role in prey capture through hydrodynamic stealth. Escape-triggering water deformations from the strike can be cancelled through controlled suction. Experimental data obtained from larval clownfish agree with model results, suggesting that the primary role of suction in early larvae is providing hydrodynamic stealth rather than capture.

## 1. Introduction

Zooplankton serve as a primary food source for many larval marine fishes. Despite the abundance of this food, over 90 per cent of larval fish are estimated to die from starvation during the transition to exogenous feeding [1,2]. This leads to the central question of how fish larvae obtain the food needed to survive and grow? While encounter rates between the larva and its prey have been extensively investigated and modelled [3–5], encounter rate by itself cannot fully explain feeding success or failure in larval fish [6,7]. Thus, efforts have been made to account for the outcomes of encounters through analysis of the physical and hydrodynamic interactions occurring during a fish's predatory attack. Such attacks typically involve a combination of a stealthy approach, followed by a rapid strike to capture the prey [8,9]. The slow approach minimizes the hydrodynamic signals that can alert the prey and allow it to escape. When close enough, the fish launches its strike. A hydrodynamic factor arises as a fish approaches: the prey can be pushed away by the fluid displacement or ‘bow wave' preceding the advance [8,10,11]. A fish can counter this by rapidly expanding its buccal cavity, using suction to draw prey into its mouth. Indeed, suction feeding is a common mode of prey capture in older fish [11–14]. Hydrodynamic analyses of predatory attacks by juvenile and adult fish have concluded that successful captures involve a combination of ram and suction in varying proportions [14–17]. How trade-offs between these two feeding modes are regulated in larval fish is unclear. Suction is also used by larval fish [8,18]. In fact, it is widely viewed as their primary mode of prey capture [11,19,20]. However, suction in larval fish is weak due to developmental limitations [21]. We addressed these issues with the help of a unique experimental dataset of high-resolution videos of predator–prey interactions between larval clownfish, *Amphiprion ocellaris*, and the calanoid copepod *Bestiolina similis* [9,22]. This previously published video footage documents changes in the predator–prey interactions between different developmental stages of both the predator and the prey. From an analysis of the videos, a simple hydrodynamic model of the fish strike phase was developed that we used to explore capture success by varying the interacting parameters of the clownfish attack. Our goal was to determine how clownfish larvae optimize prey capture success during a period of rapid larval development and changes in prey [9]. We re-examine how strategies elucidated in older fish apply to larval clownfish.

Prey behaviour is the opposing and equally important component of the predator–prey interaction. The fish tries its utmost to capture the prey and survive, and the prey tries its utmost to thwart capture and survive. The role that parameters of the prey play in determining the outcome of an encounter is often overlooked in modelling studies. The most numerous and highly nutritious zooplankters of the world's oceans are copepods [23,24]. Many larval fish depend on them at some stage during their planktonic phase [25,26]. However, copepods are highly evasive when a water disturbance from a potential predatory threat is detected [27,28]. Water deformation from the bow wave of a predatory lunge or from suction accompanying the attack can activate sensitive mechanoreceptors on the copepod's first antennae and trigger a high-speed escape that rapidly outdistances the predator [29–31]. A complicating factor for the prey, which offers the predator a brief window of opportunity, is its neuromotor system, which imposes a reaction delay owing to nerve impulse propagation and muscle activation times [32]. All of these factors, the bow wave, suction flow, sensitivity and behavioural reaction of the copepod, have been taken into account in our model of predatory success for a larval fish.

## 2. Methods

### 2.1. Video analysis of predatory attacks

**Figure 1.**
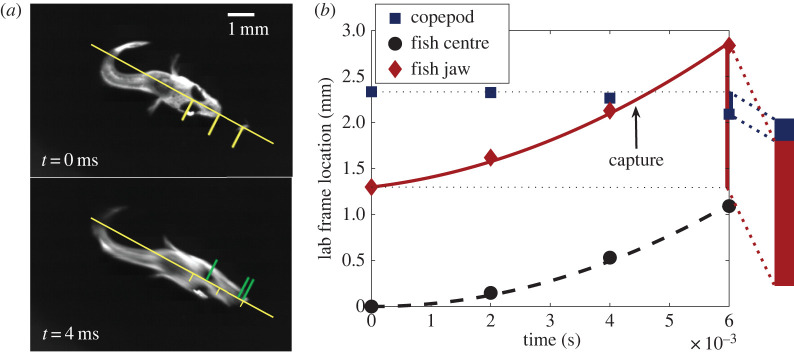
(*a*) Frames of a 7 days post-hatch (dph) fish video prior to strike (above) and immediately before capture (below), that is, the last moment the copepod's location does not overlap with the fish's mouth. Capture happens between this frame and the frame after where the copepod can be seen inside the fish's translucent mouth. Tick marks along the line of attack indicate points of interest: the point between the eyes (fish centre), the jaw's edge and the copepod. (*b*) Recorded measurements were used to fit basic curves as framework for the model. Black broken curve and the red solid curve are fitted parabolas, assuming that the fish accelerates at a constant 6.3 × 10^4^ mm s^−2^. Vertical bars at time *t =* 6 ms indicates the change in location of the fish jaw and copepod during the strike.

As a basis for model development, a collection of videos of larval *A. ocellaris* feeding on various developmental stages of the copepod *B. similis* were analysed. These videos were produced by Robinson *et al*. and deposited in the BCO-DMO public database [9,22]. Videos that had successful captures, with no attempt of escape by the copepod, were used to determine the distances covered by the fish and copepod during an attack and to get an estimate of the parameters for the model. Figure 1*a* shows a typical strike, with the starting position of the fish in the *t =* 0 ms image, and the near-capture point at *t =* 4 ms. The locations of the centre point between the eyes, the mouth (at the leading edge of the jaw) and the copepod are indicated with marks along the diagonal line of advance. The position of those and additional points at 2 ms intervals are plotted in figure 1*b*.

**Figure 2.**
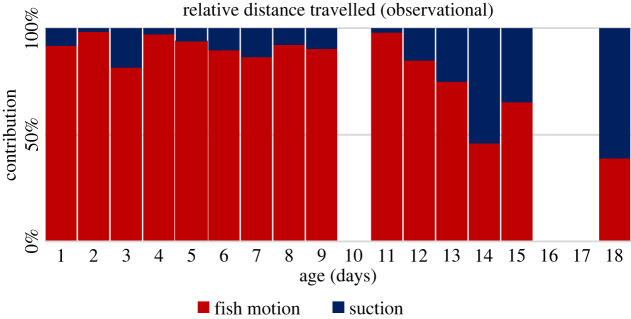
Video observations of 29 larval fish videos recording the distance travelled by both the fish and the copepod. The observed displacement of the copepod (towards the fish), *D*_prey_, was summed with the distance travelled by the fish's jaw, *D*_predator_, and the percentage of the combined distance covered by the fish *D*_predator_/(D_predator_ + *D*_prey_) was plotted (red). Data were then segregated by age group of the fish and averaged.

The total distance travelled by the fish jaw and the net negative distance travelled by the copepod from the start of the strike to the frame after capture were recorded (figure 1*b*). Then the ratio of the fish jaw movement and the absolute sum of the two distances was considered. Comparison of this ratio between different-aged fish shows an increased reliance on suction as the fish ages (figure 2).

### 2.2. Model formulation

**Figure 3.**
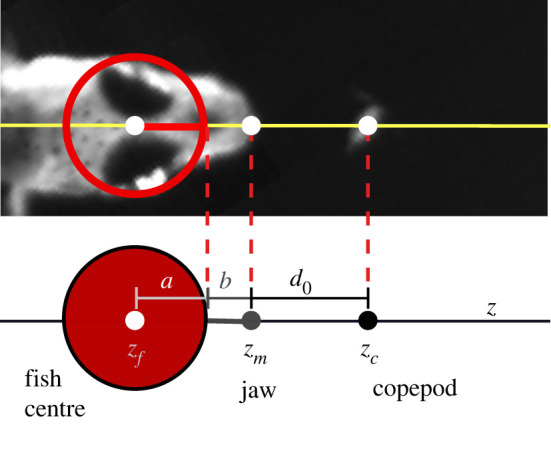
Schematic of the model. The fish is represented as a sphere of radius *a* centred at *z_f_,* with a point of suction located at a distance *a* + *b* away at *z_m_*. The initial ‘strike distance' between the suction point and the copepod's location *z_c_* is denoted by *d*_0_.

To better understand the effects of varying suction strength and other parameters, we developed a simplified model of a fish generating suction and moving directly towards a copepod. The model represents the head of the fish as a rigid sphere, a simple shape that is commonly used to represent complex bodies in animal behaviour [33,34]. Following a previously adopted approximation, the sphere is centred at the point between the eyes and has a characteristic radius *a* set by the half-width of the fish [34]. The sphere is assumed to accelerate at a constant rate *k_u_*. A single point sink of suction is located at a distance *b* ahead of the leading edge of the sphere to represent the mouth, which is in general more than one head radius in front of the fish's eyes due to jaw protrusion (figure 3). While the model approximates the fish in this manner, it does not account for any associated changes in shape of the fish's head during the strike. The fish's head size and jaw protrusion produced relatively minor effects on the results, as we discuss by tuning parameters *a* and *b* in §3.2. Suction strength, measured in units of the volumetric flow rate, is assumed to increase steadily at rate *k_m_*, starting from time *t* = 0. This assumption was partly motivated by our observation that the fish behaviour changes from displaying no suction to some suction during the short interval of the strike. The model was designed to elucidate the basic effects of ram and suction on capturing evasive prey, without incorporating the detailed shape and dynamics of the fish.

The model considers a freely suspended copepod, initially at a distance *d*_0_ from the mouth. The copepod follows the surrounding fluid flow generated by the fish, despite its minor density difference with the fluid, because it remains nearly stationary and experiences minimal acceleration for most of the duration of the strike. To predict the velocity field of the flow ahead of the fish, we adopt potential theory for inviscid flow [35], which neglects the viscous boundary layer on the rapidly accelerating fish. The boundary layer evolves with a characteristic thickness, which is estimated by considering a body moving steadily at the maximum speed [33]. The thickness of the boundary layer is expected to be very thin, of the order of *a*/Re^1/2^ [35], where the Reynolds number Re = *UL*/υ ∼ 200 is estimated using the characteristic length *L* = 2*a* ∼ 1 mm, velocity *U* = *k_u_t* ∼ 200 mm s^−1^, given by the typical acceleration *k_u_* ∼ 4 × 10^4^ mm s^−2^ over a duration of *t* ∼ 6 ms for a typical fish and kinematic viscosity of seawater υ ∼ 1 mm^2^ s^−1^. The Reynold number had a range of 120–490 for fish within days post-hatch (dph) 1–14. Thus, we expect the copepod to remain outside of the influence of the fish's momentum that diffuses through the surrounding fluid due to viscosity. At any position *z* along the direction of motion of the sphere, the velocity of the inviscid flow is given by

2.1 $$u=\frac{dz}{dt}=\frac{Ua^{3}}{{\left(z-z_{f}\right)}^{3}}-\frac{M}{4\pi {\left(z-z_{m}\right)}^{2}},$$

where *U* = *k_u_t* represents the velocity of the sphere at time *t*, *z_f_*(*t*) = (1/2)*k_u_t*^2^ represents the position of the centre of the sphere, *M* = *k_m_t* represents the suction strength, and *z_m_*(*t*) = *z_f_*(*t*) + *a* + *b* represents the point of suction. The velocity is used to predict the position of the copepod, *z* = *z_c_*(*t*), given the initial value, *d*_0_ + *a* + *b*. The maximal deformation rate is defined as the maximum magnitude of the eigenvalues of the rate-of-strain tensor [33,36]. As the model assumes that the fish makes a direct approach to the copepod, the maximal deformation rate is simply the magnitude of the gradient of *u* in the *z-*direction in the axisymmetric fluid flow around the copepod. Thus, the maximal deformation rate is given by

2.2 $$\;\left|\frac{\partial u}{\partial z}{|}_{z=z_{c}}\right|=\left|-\frac{3Ua^{3}}{{\left(z_{c}\;-\;z_{f}\right)}^{4}}\;+\frac{M}{2\pi {\left(z_{c}-\;z_{m}\right)}^{3}}\right|,$$

which is used to predict the detection time, *t_d_*. We set the detection time to be when the maximum deformation rate reaches a threshold value, *k_d_*, which represents the sensitivity of the copepod. To this, a constant reaction-delay time, *t_r_*, is added to give the calculated escape time, *t*_esc_. The model also calculates the capture time, *t_c_*, defined as the time at which the fish's mouth is predicted to contact an unresponsive copepod. The model then registers a successful capture if *t_c_* ≤ *t*_esc_, indicating that the fish made contact within the allotted time. Additionally, the model registers the attempt as a failure if the capture time exceeds the calculated escape time. To prevent the model from running indefinitely, we set an upper time limit of 10 ms, which is longer than the duration of all strikes considered in this study. Attempts lasting more than 10 ms are registered as a failure in our model.

**Figure 4.**
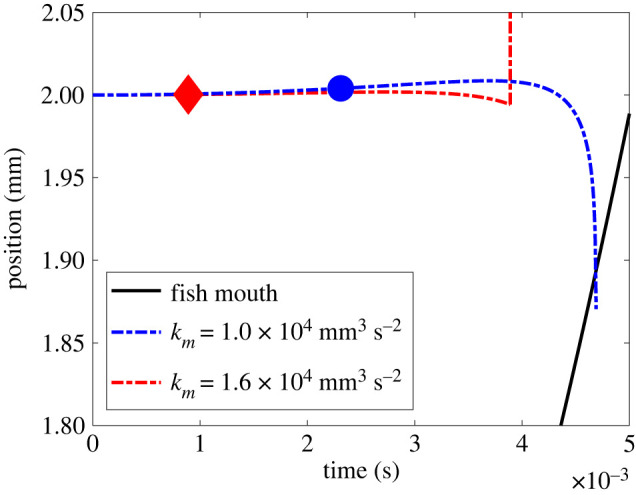
Two model examples with different outcomes. Broken blue and red lines show copepod's location while the black solid line represents the fish's mouth position. Points marked by symbols represent times when the deformation rate of the water around the copepod surpasses the set threshold for reaction, *k_d_* = 1 s^−1^. In both cases shown, the fish accelerates by the same amount, *k_u_* = 6.3 × 10^4^ mm s^−2^, and all other parameters are given in table 1. The red line shows the trial where a fish has a suction increase rate of *k_m_* = 1.6 × 10^4^ mm^3^ s^−2^, alerting the copepod at time *t* = 0.9 ms (red diamond) and subsequently fails to capture before the copepod reacts (*t_r_*) 3 ms later. The blue line shows the case of suction increasing at a rate of *k_m_* = 1.0 × 10^4^ mm^3^ s^−2^, which results in a more delayed alert time of *t =* 2.3 ms (blue circle) and does result in capture before the escape can occur.

Capture and escape outcomes are demonstrated in figure 4, which shows the position of the copepod (blue and red curves) in two separate simulations, differing only in the suction strength of the fish's mouth. The fish's acceleration is fixed, as shown by the common solid black curve. With the stronger suction, the threshold deformation rate at the copepod is reached in less than 1 ms (red line and diamond), and the copepod escapes after its reaction delay 3 ms later before the fish's mouth reaches it. In the second case, with a gentler suction, the fish has a chance to get closer to the copepod before, at approximately 2.5 ms, the latter's detection threshold is reached, but the fish's mouth reaches it before its 3 ms reaction delay allows it to escape, so it is captured (blue line and circle).

### 2.3. Model parameters

The model was parametrized using published experimental data referenced in §2.1 [9,22]. In general, specific values for attacks varied substantially, especially among different age-classes of fish [9]. Thus, mean values for these parameters were segregated into three age-classes in table 1 and their averaged values used as base parameters in the model. The parameters *a*, *b* and *d*_0_ shown in figure 3 were estimated as described above, using images of the fish and the copepod just prior to the strike. The body ram, *k_u_*, was determined by a least-square fit of the centre of the fish to the curve *z_f_* = (1/2)*k_u_t*^2^.

**Table 1.** Average parameters taken from video footage, grouped by fish age class in days post-hatch (dph). Symbols for each parameter are referenced in the model details. Value *n* indicates the sample size of values. Age classes are those identified by Wittenrich & Turingan [37] based on morphology and predatory effectiveness as confirmed by Jackson & Lenz [38] and Robinson *et al.* [9].

parameter	symbol	units	model value	dph 1–4 *n* = 8	dph 5–9 *n* = 9	dph 10–14 *n* = 4
suction acceleration	*k_m_*	mm^3^ s^−2^	1–10^6^	1.9 ± 1.3 **×** 10^3^	3.9 ± 3.1 **×** 10^3^	1.4 ± 1.1 × 10^5^
ram acceleration	*k_u_*	mm s^−2^	1–10^6^	3.6 ± 2.0 **×** 10^4^	4.0 ± 2.0 **×** 10^4^	5.7 ± 4.5 × 10^4^
body radius	*a*	mm	0.7	0.56 ± 0.03	0.65 ± 0.09	0.79 ± 0.07
suction offset	*b*	mm	0.5	0.42 ± 0.09	0.58 ± 0.10	0.66 ± 0.14
strike distance	*d*_0_	mm	0.8	0.60 ± 0.23	1.0 ± 0.30	0.88 ± 0.26
sensitivity threshold	*k_d_*	s^−1^	1			
reaction delay	*t_r_*	s	0.003			

Rate of increase of the suction, *k_m_,* was estimated by a series of trial simulations, ensuring that the copepod's simulated position matched the location observed in the last frame prior to capture. The procedure relied on an iterative method, beginning with initial guesses for the lower and upper bounds for *k_m_*, and then running the simulation with *k_m_* set at the midpoint. Either the lower or upper bound was then moved to that midpoint, depending on whether the copepod's simulated position was greater or less than that observed. This continued until the two bounds were within 0.02 log units of each other.

With a collection of proposed *k_m_*, *k_u_* pairings, we look at some commonalities between fish of similar age groups. Additionally, these pairings were compared with model runs of the corresponding age-group model fish varying in *k_d_.* Since copepod sensitivities to escape-triggering deformation vary and range from 0.4 to 6.0 s^−1^ [36,39–41], a representative mid-range value was used as a base. One must also consider the unavoidable response lag of the copepod, *t_r_* = 3 ms in our model. This stems from the copepod's nerve impulse propagation and muscle activation times [32]. The time frame varies from around 2–3 ms for small paracalanid copepodids to 4–5 ms for nauplii [39]. Table 1 summarizes the findings and the corresponding values used in the basic model. The model has seven dimensional parameters, which could be reduced to five dimensionless parameters after rescaling all lengths and times. We keep the parameters dimensional with the units given in table 1.

## 3. Model results

### 3.1. Basic model properties

**Figure 5.**
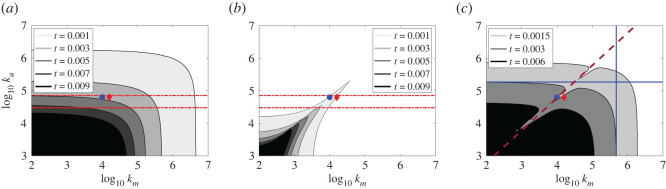
Results from the basic model using parameters from table 1. (*a*) Times for capture, *t_c_*, of a free-floating passive particle positioned at an initial distance of 0.8 mm in front of the mouth of the model fish. (*b*) Times, *t_d_*, for water deformation rates from the strike surpassing the threshold *k_d_* = 1.0 s^−1^*.* Broken lines bracket of (*a*) and (*b*) indicate experimentally observed range of fish peak accelerations [9]. (*c*) The time difference between (*a*) and (*b*), where contours represent the boundaries between a capture zone (*t_c_* < *t*_esc_) and an escape zone for different values of the copepod's reaction-delay time, *t_r_*. Solid lines indicate the theoretical requirement of capture solely relying on acceleration, *k_u_* = 1.78 × 10^5^ mm s^−2^ (equation (3.1)), or solely on suction *k_m_* = 4.77 × 10^5^ mm^3^ s^−2^ (equation (3.2)) given the parameters in table 1. Dashed diagonal line indicates the approximate linear relationship of *k_m_* and *k_u_* that maximizes the time under deformation threshold, *k_u_* *=* 6.04 *k_m_* (equation (3.4)). The blue circle and red diamond indicate parameter combinations for the corresponding symbols in figure 4.

To first see which parameter combinations result in capture, a series of trials was run using the base parameters given in the ‘model value' column of table 1. These trials vary only in *k_m_* and *k_u_* between equally spaced log_10_ units. Plotted in figure 5 are the predicted times for unresponsive-copepod capture, copepod detection and copepod escape (*t_c_*, *t_d_* and *t_c_–t_d_*, respectively) as two-dimensional heat maps. The initial set of runs was made without the copepod's escape to provide a baseline of the fish's ability to capture passive free-floating particles (figure 5*a*). The black sector at the lower left represents predicted capture times greater than 9 ms, the upper limit observed in experiments [9]. In general agreement with other studies, the range of observed ram accelerations from table 1 lies within 3 × 10^4^–7 × 10^4^ mm s^−2^, indicated by the limits marked by the broken lines in figure 5*a* [9]. Within this range, the theory predicts capture times mostly below the 10 ms mark as expected. The figure shows that, when either ram or suction is minimal (small *k_u_* or *k_m_* values), the time to capture becomes insensitive to the small parameter. Thus, for pure suction, this is the time it takes the fish to draw in a spherical volume of water of radius *d*_0_, which produces the parallel vertical bands that are functions of *k_m_* only. For pure ram, the contours form parallel horizontal bands corresponding to times different ram accelerations require to reach the particle at its original position augmented by a small displacement caused by the bow wave (roughly 2% extra distance to be covered in the basic model). The added displacement is small because the bow wave decreases with the cube of the distance in front of the sphere centre and is thus substantially attenuated by the extension of the jaw. When ram and suction are both employed and *k_m_* is roughly three-quarters of a log unit above *k_u_*, the same capture time could be achieved at a modestly lower value for each parameter, leading to the rounded corners in the figure.

Figure 5*b* displays a heat map of the predicted detection times, *t_d_*, when the deformation rate at the copepod surpasses a threshold of *k_d_* = 1.0 s^−1^ for different combinations of *k_m_* and *k_u_*. Once so alerted, the copepod will escape if the mouth of the fish fails to reach it by the end of the copepod's reaction-delay interval *t_r_* (3 ms in the basic model)*.* In cases where the attempts are mainly carried by suction or ram, models using the basic parameters predicted short detection times: under a millisecond for *t_c_* < 10 ms (comparing figure 5*a*,*b*), requiring a strong effort by the fish to succeed in capture. However, of prominence in the *k_m_*, *k_u_* heat map is a ‘ridge' of delayed detection that emerges from a combination of more modest ram/suction values (figure 5*b*). This is owing to the partial cancellation of the deformation rates from the compressive bow wave and the expansive suction. The peak of this cancellation ridge follows a slope of 1 in the log–log plot, which indicates that changes in suction and ram must be in the same proportion to maximize the detection time that the cancellation produces. Such a cancellation has been noted in the literature as creating a potential ‘sweet spot' for the approach strategy of a fish to avoid detection by the copepod [42]. The model demonstrates that this cancellation region exists for the rapid strike as well and might be exploited by a fish to improve its capture success.

The algebraic difference between corresponding (*k_m_*, *k_u_*) points on figures 5*a*,*b* (*t_c_*−*t_d_*) is shown in figure 5*c*. For each pair of *k_m_* and *k_u_* values, it gives the minimum reaction-delay time, *t_r_*, that the copepod must have to escape. For longer delays, capture by the fish is successful. Assuming equal reaction delays, a ‘capture boundary' for the basic reaction delay of 3 ms (table 1) can be created to show successful *k_m_*, *k_u_* pairs. The capture boundary divides such plots into two zones, an ‘escape zone' in the lower left corner and a ‘capture zone' covering the remainder. The capture zone above the boundary represents captures dominated by the ram capabilities of the fish; while the zone to the right represents ‘suction-dominated' captures.

The horizontal and vertical lines in figure 5*c* represent analytic computations obtained in the ram-dominated and suction-dominated regions for the reaction time of the basic model (3 ms, table 1). In the ram-dominated region, the acceleration needed for the mouth of the model fish starting at a distance *d*_0_ to reach the initial position of the copepod within the duration of the reaction time is given by

3.1 $$k_{u}=\frac{2d_{0}}{t_{r}^{2}}.$$

Although the copepod's minor displacement and delay in detecting the bow wave are neglected in this analysis, equation (3.1) agrees reasonably well with the nearly horizontal capture boundary in the upper left corner of figure 5*c*. In the suction-dominated region (no ram), the differential equation (2.1) with *U* = 0 can be solved analytically to obtain the threshold suction rate

3.2 $$k_{m}=\frac{8\pi }{3t_{r}^{2}}d_{0}^{3}.$$

This agrees with the nearly vertical capture boundary in the lower right corner of figure 5*c*. Equations (3.1) and (3.2) offer approximate conditions for the fish to capture with either ram or suction only, assuming the prey at initial distance *d*_0_ escapes in time *t_r_*.

Thus, the model can be used as a predictor of predator effort needed to effect capture of a copepod species with known *t_r_*. An indentation emerges along each capture boundary, representing a region of facilitated capture, where the contrasting deformation rates from bow wave and suction cancel each other out. This is the manifestation of the ‘sweet spot' in the relations between the accelerations in ram and suction that can reduce the fish's effort needed for a successful capture. To estimate suitable *k_m_*, *k_u_* pairings in the cancellation region, the deformation rates of early times were considered. With this assumption, equation (2.2) can be approximated by

3.3 $$\frac{\partial u}{\partial z}=\left(-\frac{3k_{u}a^{3}}{z^{4}}+\frac{k_{m}}{2\pi {\left(z-a-b\right)}^{3}}\right)t+O(t^{3}).$$

Equation (3.3) implies that the deformation rate around the copepod remains negligible initially provided that

3.4 $$k_{u}=\frac{{\left(a+b+d_{0}\right)}^{4}\;}{6\pi a^{3}d_{0}^{3}}k_{m},$$

which is obtained by setting *z* = *a* + *b* + *d*_0_ and (∂*u*/∂*z*) = 0 in equation (3.3). Equation (3.4) is plotted as a dashed line in figure 5*c* and is consistent with the general slope of the cancellation region predicted by the model.

### 3.2. Effects of parameters on model

**Figure 6.**
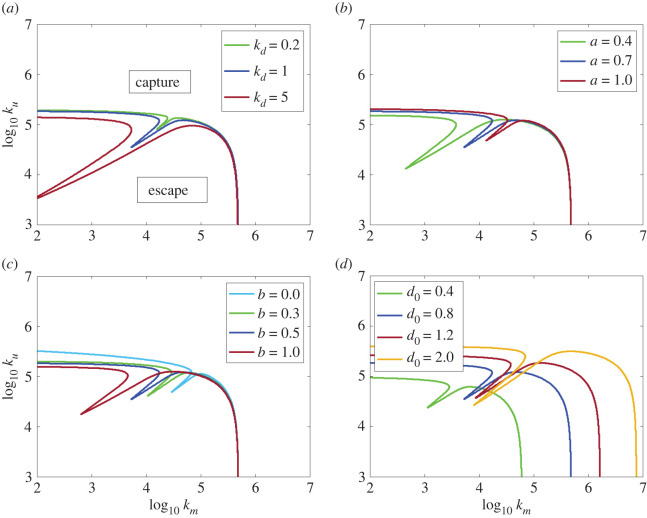
Effects on capture boundaries of altering different model parameters where *k_m_, k_u_* pairs above the lines result in capture. (*a*) Adjusting the deformation-rate threshold, *k_d_* (s^−1^), alters the size of the region of deformation cancellation. (*b*) Altering the radius of the sphere, *a* (mm), used to simulate the fish head, as is seen during the growth of the fish. (*c*) Alterations on the jaw-protrusion parameter, *b* (mm). (*d*) Alterations on the starting distance between the fish mouth and the copepod, *d*_0_ (mm). Dark blue line indicates the boundary shown in figure 5 for comparison.

The fixed parameters of the basic model (table 1) were varied to better understand their effect on capture success. For any given *k_m_, k_u_* pair, alterations in copepod sensitivity, *k_d_*, within experimentally observed ranges altered the detection time, *t_d_*, with a consequent effect on the capture time, *t_c_* and hence the curve describing the capture boundary (figure 6*a*). As mentioned in §2.3 of the Methods, *k_d_* determinations may range from 0.4 to 6 s^−1^ [36,40]. Higher *k_d_* values produced a greater indentation in the capture boundary, suggesting that the amount of stealth that bow wave cancellation by suction can confer is greater for insensitive copepods.

The effect on the capture boundary of adjusting parameters related to the size, shape and strike distance of the larval fish is also shown in figure 6. From figure 6*a*, adjustment of copepod deformation threshold causes a larger range of successful capture parameters while not affecting the overall shape of the capture boundary. Figure 6*b* shows that increasing the size of the sphere leads to stronger reliance on suction for stealth captures. Alternatively, the adjustment of the sphere can be used to estimate the error in hydrodynamic equivalence of the sphere versus the fish size. Tuttle *et al.* [34] estimated this to be ±40% based on particle tracking around swimming fish. Presented in figure 6*b* is the capture boundary, setting radius *a* to the mean radius for a mid-ranged fish (table 1), along with capture boundaries with a 40% difference in the mean radius. The primary effect of this equivalence error is to change the size and position of the cancellation region. It has little effect on the capture boundary at the extremes of low suction or low ram. Figure 6*c* shows the effect of different amounts of jaw protrusion beyond the radius of the sphere. While the jaw (bearing the mouth at its distal end) in this case is fixed (in real fish it is protruded early in the attack), it allows the fish to launch its attack with its bow wave-producing body at a greater distance from the prey, which decreases the likelihood of being detected. It also has the effect of placing the suction sink further ahead of the bow wave, and as can be seen from the figure, this deepens the indentation of the capture boundary, enhancing the level of deformation cancellation possible. Figure 6*b*,*c* shows that the parameters *a* and *b* have minimal impact on the capture boundaries, except for the shift in the cancellation region. This suggests that, over most of the range modelled, the fish's head size and jaw protrusion play a relatively minor role in determining fish capture. Figure 6*d* shows the effect of having the fish launch its attack from different strike distances. Decreasing this distance substantially reduces the accelerations of pure ram or pure suction needed for capture. This also shifts the capture-facilitation region due to cancellation of opposing deformations while keeping the minimum acceleration needed for capture unchanged.

## 4. Comparison with experimental observations

**Figure 7.**
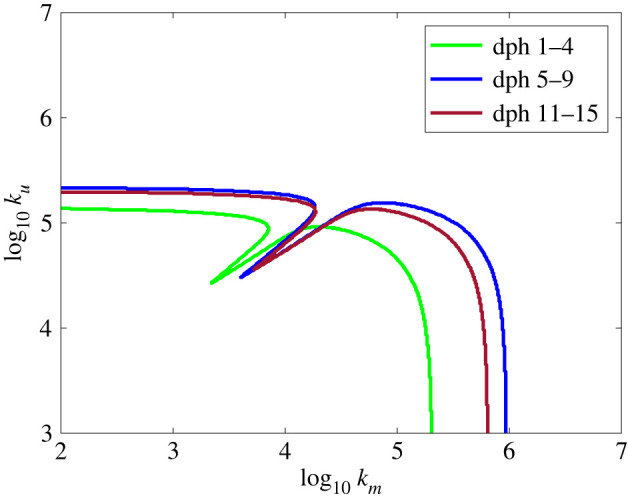
Model simulation of the three larval fish age groups (table 1).

This section addresses how well the model predictions correspond to experimentally observed attacks. Figure 7 shows capture-boundary plots for three age groups of fish using the fish parameters for each age group shown in table 1. Copepod parameters remained constant through all runs. There is a rightward shift in the cancellation zone with fish age, suggesting the potential benefit of relying more on suction than ram as the fish ages (figure 2). Also worth noting, the parameters used for dph 5–9 have the highest effort requirement for both pure suction and pure ram attempts, consistent with the attacks being launched from greater distances (*d*_0_).

**Figure 8.**
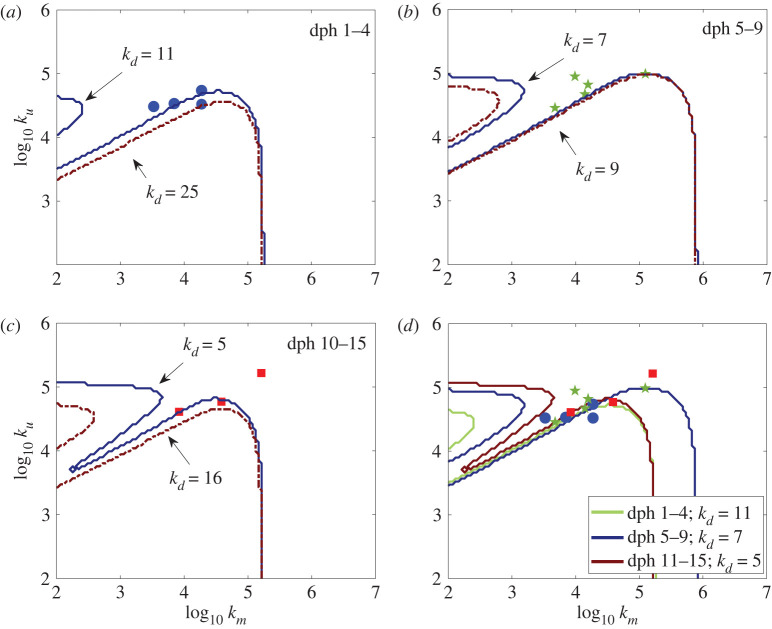
Best-fits for *k_m_* and *k_u_* with other parameters of the model set from specific observed encounters ending in capture. *k_d_* values adjusted to produce agreement with observed captures so the experimental points fall into the capture zone of the plots. (*a*)–(*c*) Points of fish data from each of the age groups (table 1). Model parameters were set individually from each observed encounter, then two sets of simulations were run at *k_d_* increments of 1.0 s^−1^: one set was run until all but one point fell within the capture zone (solid blue curve), the second was run until all points fell within the capture zone (broken red curve). (*d*) Comparisons between the blue lines from (*a*)–(*c*).

Using the age-specific parameters given in table 1, trials were run with varying *k_d_* values to approximate the *k_d_* value needed for capture using fitted *k_u_* and estimated *k_m_* values from specific observations (figure 8). While any non-zero set of *k_m_*, *k_u_* parameters can result in model capture by setting the copepod's deformation threshold *k_d_* to a sufficiently high value, the minimum *k_d_* value required for the measure *k_m_*, *k_u_* sets is within the same order of magnitude as those reported in the literature. This shows that the model is consistent with the experiments. However, the actual deformation threshold of the observed copepods may vary because the capture zone depends sensitively on the choice of *k_d_* (figure 6*a*).

## 5. Discussion

We have presented a simple hydrodynamic model to examine the relative contributions of ram and suction to successful prey capture by clownfish larvae. This model can be used to explore optimal strategies for capturing evasive copepod prey, as the prey's ability to sense strike-caused hydrodynamic disturbances and the near guarantee of a prey's escape, if attempted, are both considered. The model suggests that contrary to expectations, ram feeding is the dominant mode for early larvae. Nevertheless, weak suction in early larvae may be a critical part of the predatory strategy by producing a region of hydrodynamic stealth during the strike.

### 5.1. Impacts of the model's simplifications

The model presents a new conceptual framework for analysing capture-escape boundaries between larval fish and evasive copepod prey. The model not only predicts the existence of the cancellation region, but it also demonstrates how this region changes with predator or prey parameters. Cancellation arises when the two competing effects of ram and suction produce nearly equal and opposite deformations around the prey, which is essential for delaying the copepod's detection in the early phase of the strike. During this critical phase, when the fish is still distant from the copepod, the shape of the fish's mouth and the effects of viscosity are expected to remain negligible. Unlike larval seabream suction feeding at low Reynolds numbers [2], which led China and Holzman to conclude that water viscosity decreases first-feeding success and results in ‘hydrodynamic starvation', the larval clownfish in our study are expected to produce nearly inviscid flow through rapid acceleration. Nevertheless, in the final milliseconds prior to capture, when the mouth is near the copepod, we expect the shape of the fish's mouth and the effects of viscosity to become more important. The neglect of these factors is a limitation of our model.

Our model of larval fish feeding was designed to elucidate the basic effects of suction and ram on evasive prey. Fish feeding is a highly dynamic and complex process depending on morphological, behavioural and hydrodynamic factors [11,43]. Early models represented the effect of suction feeding fish on the far-field flow by a moving point sink [44] and a vortex ring [45], while the near-field flow depends on subtle changes in the shape of the fish's body and mouth opening [46,47]. A more recent model incorporated evasive prey in a suction flow generated by a stationary point sink to account for fish relying predominantly on suction [10]. However, some predators rely on approaching the prey for feeding, as modelled at both high [33] and low Reynolds numbers [48] without incorporating suction or prey. Our model treated evasion-triggering sensitivity in a highly simplified manner, characterizing it by a single parameter, *k_d_*. Other models have gone beyond this, wherein the flow detection by evasive copepods has been modelled to different degrees, down to the scale of a single mechanosensory hair in a simple shear flow that oscillates with time [49]. Our study builds on previous studies by incorporating a combination of ram and suction by larval fish while they attempt to capture evasive prey.

### 5.2. Insights from the study

#### 5.2.1. Suction is not the primary capture mode in early larval clownfish

The widely held view that larval fish capture prey by suction was tested here experimentally and with the model [21]. Video observations showed little evidence for the expected displacement of a passive copepod toward the mouth of the attacking first-feeding larval fish [19]. The copepod hardly moved, even when visible within the fish's translucent mouth. Holzman & Wainwright [10] suggest that for suction-dependent capture, the suction must be initiated before the bow wave from the ram alerts the copepod, since once an escape commences, the copepod becomes much harder to capture. As a strategy for ‘how to surprise a copepod', they propose that the fish launches the body ram *before* opening its mouth to exert sudden suction to overwhelm the copepod before it can react to the ram cue. Although early *A. ocellaris* larvae lack the ability to generate overwhelming suction, the model predicts that in pure ram attacks, the copepod would experience water deformations that its mechanoreceptors can detect within a fraction of a millisecond of the launch of the attack (figure 5*b*). The fish must thus employ an alternative strategy for a successful capture.

#### 5.2.2. Hydrodynamic stealth: escape-triggering by the bow wave can be cancelled by suction

Clownfish excel in stealth as a strategic alternative to strong suction. Their approach to copepod prey can take two orders of magnitude longer than the strike, producing a much-reduced hydrodynamic disturbance for detection by the copepod [9,34]. In addition, the model suggests that stealth can be provided by deformation cancellation. A similar cancellation of bow wave by suction was proposed some years ago in studies on other aquatic predators (e.g. [50]) and has been demonstrated experimentally by Gemmell *et al.* [42] using particle-imaging velocimetry to study the approach phase of adult zebrafish to copepod prey. The fish were shown to use ‘compensatory suction' to cancel the deformation from the bow wave generated by the approaching fish. Our model predicts such a cancellation is possible as well for the much more rapid strike phase of the clownfish, when the compressive deformation from the bow wave is locally matched by the expansive deformation from suction (figure 5).

The cancellation forms a narrow ‘indentation' in the capture boundary of ram–suction parameter space, a region of facilitated capture that might be exploited by a planktivorous fish. However, to exploit this region requires coordination between ram and suction on the part of the fish. Experimental data suggest that the larval fish may have this skill. Using parameters set from observed strikes, data points align intriguingly close to this cancellation region (figure 8). Thus, the energy expended for capture can be reduced if the fish can produce results in this region. A significant result from studying this cancellation region is that it is much more prominent for less-sensitive copepods, that is copepods with high minimum deformation-detection thresholds. If the deformation threshold of a copepod in the model is set to values in the lower end of the range observed experimentally (0.1–1 s^−1^), the cancellation region becomes very small (figure 6*a*). For the cancellation region to be significant, the deformation threshold of the copepod predicted by the model must be much higher than the thresholds that correspond to the sensitivities measured experimentally.

#### 5.2.3. Predicted sensitivity of copepods to the rapid strike is significantly lower than expected

For its survival, the copepod relies on high sensitivity to water deformation to give timely warning of a predatory attack. A wide range of values for the minimum escape-triggering deformation rate has been reported from 0.04 s^−1^ for slow-approaching predatory fish to 0.4–20 s^−1^ for artificial suction tubes [34,36,51]. However, the suction tube, commonly relied on for estimating sensitivity, generates deformation rates that rise very slowly compared with those from a fish strike, limiting their applicability to strike sensitivities [32]. Sensitivities to rapidly rising rates akin to those produced by body ram have been studied experimentally using abrupt movements of nearby solid bodies. These studies report deformation thresholds ranging from means comparable to those reported for suction tube experiments (over 15–0.4 s^−1^ among different free-swimming calanoid species [40,41]) down to astonishingly low deformation thresholds of 0.002–0.015 s^−1^ for rapid-rising flows at antennular tips of tethered animals [32,52]. However, our model suggests that a successful copepod capture for a fish accelerating at a rate comparable to experiments (broken lines in figures 5*a*) requires that the copepod's deformation threshold to be set to the upper limits of the reported range. Behavioural responses to predator mimics are variable despite controlled experimental conditions, and decreased sensitivities may occur if the copepod is engaged in feeding or if the orientation of the copepod is non-optimal [53,54]. The actual sensor-activating flow produced by the deformation depends on the orientation of the copepod's sensor-bearing first antennae, which can result in orientation-related ‘blind spots' in sensitivity that can render the copepod less sensitive than the experiments would suggest [54].

#### 5.2.4. Milliseconds matter

Timing is a key factor in the success or failure of a strike, as few larval clownfish succeed in capturing a copepod once an escape is initiated [9]. The model predicts that acceleration of the fish in the observed range produces deformation rates that grow very rapidly at the close distances from which the fish launches its attack (figure 5*b*). This sets up a race between the copepod's response-time and the speed of the lunge. Two conclusions can be drawn: (i) to succeed in capture, the fish must reach the copepod before it initiates its escape and (ii) the copepod can increase the probability of escaping capture by minimizing the delay of its behavioural escape response. The impact of this balancing act is apparent from the relatively large effect that changes in the reaction time make in the required fish performance (figure 5*c*). Thus, the model quantifies how critical the early moments of the strike are for a successful capture.

#### 5.2.5. Future steps

An informative extension to the current study, using the same publicly available data, would be to map the strike parameters leading to escape. It would be of interest to know whether a study akin to that in figure 8 would predict a similar lack of detection sensitivity, *k_d_*, for the copepod. The model results also lead to two complementary directions for future research. The model identified behavioural elements in the larvae's predatory strategy that maximize strike effectiveness while minimizing detection by the prey. However, the prediction of a cancellation zone needs to be confirmed through empirical experiments on clownfish and other fish larvae. The goal of such studies would be to parametrize a generalized model of predator–prey interactions that would inform ecological studies and models by predicting which fish larvae prey on copepods and which need to rely on non-evasive prey. Another research direction is the refinement of the model—these predator–prey interactions occur in a hydrodynamic environment that is characterized by viscous and inertial forces, which are rapidly changing over very short time scales (milliseconds). Future modelling efforts need to focus on the complex fluid flow by incorporating the dynamics and morphology of the predator and prey.

## Data Availability

Raw numerical data and producing MATLAB codes are provided in the electronic supplementary material [55]. Video supplying the raw numerical data was sourced from publicly available data found at Biological and Chemical Oceanography Data Management Office [22]: https://www.bco-dmo.org/dataset/747926.
